# Molecular Programming of Mesodiencephalic Dopaminergic Neuronal Subsets

**DOI:** 10.3389/fnana.2017.00059

**Published:** 2017-07-19

**Authors:** Marten P. Smidt

**Affiliations:** Molecular NeuroScience, Swammerdam Institute for Life Sciences, University of Amsterdam Amsterdam, Netherlands

**Keywords:** dopamine, differentiation, specification, development, mesencephalon, molecular coding

## Abstract

Dopamine neurons of the substantia nigra compacta (SNc) and ventral tegmental area (VTA) are critical components of the neuronal machinery to control emotion and movement in mammals. The slow and gradual death of these neurons as seen in Parkinson's disease has triggered a large investment in research toward unraveling the molecular determinants that are used to generate these neurons and to get an insight in their apparent selective vulnerability. Here, I set out to summarize the current view on the molecular distinctions that exist within this mesodiencephalic dopamine (mdDA) system and elaborate on the molecular programming that is responsible for creating such diversity.

## 1. Introduction

Regulation of behavior and more specific emotion is critically regulated by dopaminergic neurons located in the mesodiencephalon. These dopaminergic (DA) neurons can be sub-classified within their own groups by specific molecular features (reviewed in Smidt and Burbach, 2007; Veenvliet and Smidt, 2014; Arenas et al., 2015). Recently, these apparent molecular differences have been associated with selective vulnerability in terms of intrinsic higher dependency and/or related to higher metabolic demand (Jacobs et al., 2007, 2011; Pacelli et al., 2015; Doucet-Beaupré et al., 2016). This underscores the importance of understanding the exact molecular components that drive the creation of this diversity. Recently, the initial gap between the theoretical description of molecular distinct dopaminergic population and the actual description has been closed with the emergence of detailed single cell transcriptome data of mdDA neurons (Poulin et al., 2014; Anderegg et al., 2015; La Manno et al., 2016). Cluster analysis of such data together with in depth gene function analysis (Smidt and Burbach, 2007; Veenvliet and Smidt, 2014; Arenas et al., 2015) has shown that both in mice and humans mdDAneurons clear distictions can be made between neuronal subpopulations. These data will open up new avenues in the understanding of the relation of specific molecular programming and selective vulnerability as observed in SNc neurons. Moreover, such advances will enhance the opportunities for the development of clinically relevant cell-replacement therapies as an ultimate treatment for Parkinson's disease (PD).

## 2. Regional coding that drives mdDA diversity

A recent birth dating study (Bye et al., 2012) indicated that A9 (SNc) neurons precede A10 (VTA) dopamine neurons. This might also indicate that part of the lineage differences may rely on specific changes of ventricular zone radial glia in time to generate specific subsets of dopamine neurons, which may originate from the same position in the ventricular zone. Such a mechanism would generate essential lineages which depend on further sub-specification during their trajectory in final differentiation and migration. This notion corresponds very well with initial data on fate mapping studies with Gli1 and Shh driven Cre-ERT2 alleles and ROSA-lox-stop-lox reporters (Blaess et al., 2011). They show that the rostral/lateral positioned dopamine neurons visualized at E12.5 are initially responding (Gli1 activated) in precursors at E7.5 and start to express Shh themselves around E9. From that data-set it is also clear that the medial/caudal (VTA) DA neurons are labeled at later stages of ventricular zone development. Note that this early coding information is not directly coupled to the actual birthdating events of DA precursors. Quantitative measurements indicate that there is a continues shift of labeling of DA neurons from rostral to caudal between E8.5 and E11.5 of Shh-expressing precursors. They have substantiated this coupling by measuring subset markers such as Girk2 (SNc) and Calbindin (VTA) in both labeled DA neurons, where the early Shh expressing precursors map the Girk2 population and the late group the Calbindin population. This temporal phenomenon of Shh expression in the ventricular zone precursors might be indicative of the coding- and birth-dating differences between rostral (SNc) and caudal (VTA) DA neuronal populations, although the molecular mechanisms behind this phenomenon is not described in detail. Noteworthy, the timing of the Shh response suggests that Shh signaling does not have a role in the actual (late) differentiation program. This notion has been substantiated by an independent study where they used fate mapping of Shh induced Gli2A activation at the peak time of DA neuronal birth (E11.5; Mesman et al., 2014). Taken together, differential Shh coding seems important during the early specification phase of mdDA neuronal precursors in the ventral ventricular zone of the midbrain region.

In a few studies positional information in the ventricular zone toward lineage selection was identified by FGF signaling events and a transcriptional code within the floor plate (Lahti et al., 2012; Panman et al., 2014). Lahti et al. suggest that two principle DA neuronal populations exist in the mesodiencephalon, where the mesencephalic population depends on correct FGF signaling. Removal of this signaling induces a rostralisation effect were (immature) DA neurons adopt a diencephalic signature. Since later conditional ablation of FGF signaling, by Dat-Cre and Th-Ccre drivers, had no detectable effects (Lahti et al., 2012) the rostralisation effect in the absence of FGF signaling would rely on changes in the early specification of the region. Panman et al. described the medial exclusive expression of Sox6 next to Otx2, of which the former population develops into the SNc. In addition, through selective gene ablation they suggest that Otx2 has a repressive effect on Sox6 expression. This mechanism creates a permissive area in the medial floor plate where the SNc lineage may be formed. In the manuscript no clear rostral/caudal data is presented and therefore it is currently unknown how or whether this medial Sox6 domain is changing on that axis in the midbrain region. Nevertheless, it has been suggested that dopamine neurons originate from the midbrain floorplate until the floorplate/basal plate boundary (Mesman et al., 2014) and that the medial section contains cells that are migrating outward, which would suggest that they may form the lateral-located SNc population. These data match very well with the suggested origin of the Sox6 population at E11.5 which further strengthens that data.

## 3. Description of mdDA neuronal subsets

The first appearance of molecular differences between mdDA subpopulations became apparent in Pitx3 mutant animals (Nunes et al., 2003; Smidt et al., 2004). Since Pitx3 was present in all terminally differentiating mdDA neurons (Smidt et al., 2004; Maxwell et al., 2005) around E14.5, the selective vulnerability as observed by the absence of most of the SNc in Pitx3 ablated animals, suggested the existence of SNc specific molecular determinants. In later studies downstream targets as Ahd2 were found to be involved in this phenomenon and act through the generation of retinoic acid (RA), which was subsequently able to partly rescue the Pitx3 ablation (Jacobs et al., 2007). Subsequent studies have elaborated on the possibility of the existence of such molecular differences (Smits et al., 2006) and several TFs have been implicated in the generation of molecular diversity among mdDA neurons as discussed in more detail below. Through initial basic expression studies several molecular determinants of mdDA subsets were described as Ahd2 (Aldh1a1), Pbx1/3, Cck, Adrb2, Girk2, and Dat (Slc6a3). For most of these factors a clear distinction over the anterior/posterior axis of the mdDA neuronal pool can be made already during development (Reyes et al., 2012; Smits et al., 2013). Other studies more directed to the elucidation of molecular coding have revealed additional subset markers as Calb2, Rspo2, Sox6, Otx2, Nts, and Tle3 (Di Salvio et al., 2010a; Panman et al., 2011; Hoekstra, E. J. et al.; Veenvliet et al., 2013). In addition, it has become clear that based on physiological properties a clear distinction can be made within the mdDA neuronal pool (Roeper, 2013). Using clustering techniques to group specific electrophysiological features of all mdDA neurons it was suggested that 2 major groups exists each for the SNc and VTA, with two to four subgroups within those regions. Together with this distinction, it is suggested that such functional diversity is linked to specific projections (Roeper, 2013). The relationship between the molecular details and such physiological features, beyond the relative presence of Girk2 and Dat, remains to be determined.

A essential step into the full description of molecular description of mdDA neuronal diversity has been taken by the introduction of single cell transcriptome analyzes approaches (Poulin et al., 2014; Anderegg et al., 2015; La Manno et al., 2016; Poulin et al., 2016). Initially, an approach was used were a known set of genes was used to profile single cells from the mdDA neuronal pool (Poulin et al., 2014). From the distribution of these (96) genes (determined by known SNc and VTA expression profiles) in the isolated single cells (96 cells), the authors dissected the neuronal pool into 6 distinct subgroups (1A/B, 2A/B/C/D). Subsequent spatial expression analysis generated a link between the molecular profile and the location of these dopamine neurons. The 1A group is marked by high expression of Ahd2 and Sox6 and is located in the rostral SNc region without the tip area. The 1B group is marked by Sox6 but does not express Ahd2 and is located dorsally of the rostral SNc region. The 2A group is defined by the unique expression of Slc32a1 (GABA vesicular transporter, Vgat), among the genes tested, and locates to the medial/caudal VTA region and intermingles there with other subgroups. The 2B group is marked by Otx2, Ahd2 and Calb1 and located to the ventral/medial VTA. The 2C group is marked by Vip expression and is located in the peri aquaductal gray (PAG) and dorsal raphe (DR) area. Noteworthy, although it is known and confirmed here that the Dat is preferably expressed in rostral/ventral mdDA subpopulations, using Dat-Cre here to drive tdTomato (Slc6a3:Cre;Ai9 mice) did result in the isolation and characterization of mdDA neuronal subgroups that are very low or undetectable for Dat expression in the adult stage. The data presented did confirm initial ideas and data on subset specificity (Smidt et al., 2004; Smits et al., 2006, 2013) and were the first to use single cell approaches to determine the possible amount of subgroups present in this neuronal pool.

A second essential dataset was recently provided by using single cell RNAseq approaches in dopamine neurons from mice, humans and stem cells. Moreover, they included timeline analysis in humans and mice (human 6 weeks were matched to mouse E11.5 and human 10 weeks to mouse E14.5) to follow the developmental trajectory (Chakrabarty et al., 2012) in these two species (La Manno et al., 2016). In developmental stages, three subsets are defined marked by proneural genes and Th (DA0), expression of additional factors as Dat (DA1) and finally additional factors like Ahd2 and Lmo3 (DA2). This initial distinction is conserved between mice and humans. When focusing on the adult subsets, it was shown that 5 subgroups exist, one mapping to the SNc and 4 in the VTA region, confirming earlier data (Poulin et al., 2014). The SNc group is mainly located in a lateral position running from rostral to caudal. The more medial area consists of the 4 remaining VTA groups. Some genes are distributed over several distinctive groups as is the case for Ahd2 which is present in the SNc and VTA(2). Most enriched or unique genes for each group do exist and are as described follows: SNc: Lix1, Ptpn5, Fgf1, Nostrin, Serpine2, Kcnip3, Grik1; VTA1: Lypd1, Pou3f1, Cd9; VTA2: Otx2, Neurod6, Grp, Tcf12, Calca, Grp83; VTA3 (PAG): Vip, Cck, Cnr1, Nphp1, Chtf8 and finally VTA4: Slc32a1, Ctxn3, Etv1, Lmx1a. The 5 subgroups in the mature system and their description of 3 subgroups during development, raised the issues that possibly the specification of additional subgroups is a consequence of environmental cues rather than genetic programming initiated during development (La Manno et al., 2016).

## 4. Molecular coding that drives developmental subsets

### 4.1. Intersectional coding by Pitx3 and En1

The possible interplay between En1 and Pitx3 became apparent when transcriptome analysis on Pitx3 mutants revealed an upregulation of En1 and En2 in Pitx3 ablated mdDA neurons (Jacobs et al., 2011) and this upregulation was not due to the indirect absence of RA signaling. Initial analysis on En1 mutants indicated that they die at around birth and show severe hindbrain defects with relative subtle defects in the mdDA neuronal population (Wurst et al., 1994). Subsequent analyzes have indicated that En1 is involved in development and especially maintenance of mdDA neurons (Simon et al., 2003; Albéri et al., 2004; Sonnier et al., 2007; Alvarez-Fischer et al., 2011; Rekaik et al., 2015). Later it became apparent that part of the hindbrain phenotype together with the perinatal lethality was due to dosage differences of En2 caused by genome background differences in the used mutant strains. After several backcrosses to C57BL6J as a background strain it became possible to study adult En1 mutants which did not have a hindbrain defect (Bilovocky et al., 2003). Initial analyzes of these En1 mutants indicated that specific part of the SNc and VTA regions were affected by En1 ablation (Veenvliet et al., 2013). This neuronal loss partly mimicked the specific SNc loss as observed in Pitx3 mutants (Nunes et al., 2003; Smidt et al., 2004), suggesting possible crosstalk between these two transcription factors. Subsequent transcriptome analysis revealed that genes like Cck and Ahd2 were co-regulated by En1 and Pitx3, were the expression of Cck is depending on En1 and suppressed by Pitx3 in rostral areas, whereas Ahd2 is depending on both En1 and Pitx3 presence. When common target genes are selected, most genes are reciprocally regulated by En1 and Pitx3, which suggests that depending on the exact location the local concentration of Pitx3 and En1 in a neuron will define its local phenotype. Mechanistic analyzes on the crosstalk hinted toward a common mechanism where Pitx3 and En1 are able to influence Nurr1 expressed genes by the co-regulation of Hdac activity, where Pitx3 and En1 act as ligands to Nurr1 (Jacobs et al., 2009; Veenvliet et al., 2013). Furthermore, En1 expression itself is regulated by Pitx3 and vica versa. Finally, Pitx3 is able to regulate En1 modulators like Pbx1/3 and Tle3 (Veenvliet et al., 2013). The former Pbx1 has recently been shown to act as a promoter of Pitx3 expression, which suggest that these factors are cross-interacting at the transcriptional level as well (Villaescusa et al., 2016). Moreover in Pbx1 mutants, the expression of Pbx3 is enhanced at E12.5 (Villaescusa et al., 2016), suggesting that also Pbx3 is in this regulatory loop. Further co-regulation of Pitx3 has been suggested to be performed by Lmo3 (Bifsha et al., 2016), which is enriched in SNc neurons in mice and humans (La Manno et al., 2016). Taken together, possible local concentration differences that exist in Pitx3 and En1 expression influence co-regulators and together with those form a transcriptional program that causes clear distinctions already during development between rostral and caudal mdDA neuronal groups.

### 4.2. Intersectional coding by Sox6 and Otx2

The generation of mdDA neurons has been described in some detail concerning the essential role of Otx2 in early and late development stages (Smidt and Burbach, 2007; Omodei et al., 2008; Simeone et al., 2011). Next to the overall function in development more data has been produced that hint toward an additional function in subset specification (Di Salvio et al., 2010a). Initially, expression data hinted toward an more exclusive role in VTA neurons (Di Salvio et al., 2010b), which later turned out to be involved in the specific repression of the dopamine transporter (Dat) in a caudal set of VTA neurons (Di Salvio et al., 2010a). The authors finally suggest that Otx2 in these neurons influences the vulnerability to MPTP exposure, a mechanism possibly also influenced by the level of Dat in these neurons. The initially data were strengthened through results that described the intersectional coding by Sox6 and Otx2 in defining the SNc and VTA area within the mdDA neuronal population (Panman et al., 2014). Initially Sox6 and Nolz1 were identified in a expression screen in mdDA neurons (Panman et al., 2011). Experiments using a conditional ablation approach for Otx2 and Sox6 indicated that Otx2 is able to restrict Sox6 expression at early stages, leading to Sox6 negative neurons, opposed to Sox6 positive SNc neurons. On the other hand Sox6 ablation did not lead to changes in Otx2 expression suggesting that Sox6 is influenced downstream of Otx2. Nevertheless, the normal SNc subset phenotype is affected in Sox6 ablated animals as indicated by the lower expression of Ahd2 and increased expression of Calb2. Such gene expression shifts hint toward an overall more caudal (VTA) phenotype in Sox6 ablated animals and opposed to Otx2 ablated animals which hint toward a more rostral (SNc) phenotype. The molecular mechanisms of this intersectional coding remains to be elucidated.

### 4.3. Rspo2 defines a substantia nigra subset vulnerable to Lmx1a ablation

Lmx1a has been known for its essential role in chicken mdDA neurons and its described function in the ability to induce the mdDA neuronal phenotype in ES- and IPS-cells (Andersson et al., 2006). Initial Lmx1a mouse model data (Ono et al., 2007) from the Dreher mutation, suggested that the amount of mdDA neurons that arise during development was reduced to ~ 50% at E13.5, and some neurons mis-expressed the red nucleus markers Lim1/2. Here, no experiments were performed that suggested a change in subset specification or specific regional aberrations. In a later study a knock-out of Lmx1a and compound mutants of Lmx1a/Lmx1b were analyzed to solve any redundancy issues and to specify in detail the (late) functions of these TFs (Deng et al., 2011). Their data suggest that the medial mdDA area is mostly affected in terms of neurogenesis at E11 and the lateral domain is mostly affected by Lmx1b ablation as shown by the absence of D2 receptor expression at E13.5, although the authors do claim that also in the medial domain these mutants fail to express late differentiation markers as Th and Pitx3. Notably, they confirmed the overall loss of mdDA neurons as initially reported from the Dreher mutant, suggesting that the Dreher mutant is similar to the full knock-out. These data suggest that early during mdDA differentiation a delay or mis-coding exists as a consequence of Lmx1a ablation, next to an overall loss of mdDA neurogenic potential. In later studies that detailed the transcriptional program under the control of Lmx1a and the subsequent analysis in adults systems a more clear end-stage situation was provided on mdDA neuronal subset coding (Hoekstra, E. et al.,E). Their data showed in the adult a clear ablation of a SNc subset. This group of neurons expressed the Wnt modulator Rspo2 in normal conditions. Already at earlier developmental stages Rspo2, Calb2, and Pbx1 were ablated in rostral lateral positions. Moreover, next to this clear subset specific phenotype, a clear ablation of Nurr1 was found in rostral lateral positions in and outside the mdDA neuronal domain. The data clearly show a rostral specific molecular coding defect that precipitates in the absence of the Rspo2 population within the SNc. Moreover, the initial absence of Rspo2 may also indicated that modulation of Wnt signaling at this point during development may be an essential additional signaling event for the correct generation of parts of the SNc, as an essential feature of additional coding to create the full adult diversity. Finally, a recent study hinted toward an essential role of Lmx1a/b in the mitochondrial function in mdDA neurons (Doucet-Beaupré et al., 2016). Ablation of these factors in the adult system created axonal pathology characterized by alpha-synuclein inclusion, next to mitochondrial damage, impaired respiratory chain activity and increased oxidative stress. This analogy to PD symptoms suggests that both Lim homeodomain TFs are essential for correct SNc functioning in the adult mdDA system.

### 4.4. Ahd2 function in development and adult dopaminergic subsets

Initial data described the presence of Ahd2 (Ald1a1) enriched in the SNc of the mdDA population, were it was proposed to have a major function in local retinoic acid synthesis and was suggested to play a role in disulfiram (Antabuse) induced neurotoxicity leading to parkinsonism and catatonia (McCaffery and Dräger, 1994). In line with earlier data describing the developmental role of Ahd2 in the retina (Fan et al., 2003), it was shown that RA is able to supplement initial SNc developmental defects as observed in Pitx3 ablated mice (Jacobs et al., 2007). These data suggest that Ahd2 through RA synthesis is partly responsible for a subset specific gene regulatory profile (Jacobs et al., 2011) that is established at post-mitotic states as discussed above. The regulation of Ahd2 itself depends on the interplay of En1 and Pitx3 (Veenvliet et al., 2013), however it is currently unknown which mechanisms underlies the suppression of Ahd2 expression in specific parts of the mdDA neuronal pool.

The importance of the detoxification role of Ahd2 was underscored by analyzes on Ahd2 and Aldh2 double knock-outs, both normally present in dopamine neurons. It was shown that such mice display significant loss of TH neurons accompanied with an age dependent lower performance in motor output. Moreover, the levels of toxic dopamine breakdown products as DOPAL were significantly increased in such animals (Wey et al., 2012). The latter detoxification function was further substantiated by a study were in alpha-synuclein transgenic mice cells that do not contain Ahd2 deteriorate more compared to those which do. In addition, deletion of Ahd2 in this model increased the alpha-synuclein induced neurodegeneration, whereas Ahd2 over-expression protected against this neurodegenerative phenomenon (Liu et al., 2014). Mechanistically, this might be explained by the Ahd2 dependent removal of reactive aldehydes, as DOPAL, which otherwise may lead to excessive oligomerization of alpha-synuclein in dopamine neurons.

Recently, an unexpected function of Ahd2 was described that enhances the subset specific functionality of Ahd2 expressing mdDA neurons. In an elegant study it was shown that Ahd2 is part of a conserved synthesis pathway for the generation of gamma-aminobutyric acid (GABA) (Kim et al., 2015). This indicates that only in Ahd2 expressing mdDA neurons GABA is used a co-transmitter, which is modulated by ethanol. In this way ethanol influences specifically this subset of mdDA neurons in their transmission efficacy.

### 4.5. Mest is essential for the development of a SNc subset

Mesoderm specific transcript (Mest), also known as paternal expressed gene 1(Peg1), is a is a member of the divergent alpha-beta hydrolase protein family. In the liver, Mest inhibits Lrp6 glycosylation through enhancing ubiquitination of beta-catenin and therefore is able to regulate balancing of canonical and non-canonical Wnt signaling (Jung et al., 2011). In the brain Mest is expressed in the midbrain region during the mdDA developmental cycle and is still expressed in a subset of mdDA neurons in the adult stage (Mesman et al., 2017). Ablation of Mest leads to the loss of a SNc neuronal subset after the developmental stage and which is different from those cells that are lost in Pitx3 mutants. Moreover, the defect is progressive in the SNc region when comparing 3 and 8 months old animals. These defects are mirrored by the loss of Th and DA release in the striatum and reflected by the lower performance in a climbing test. Interestingly, analyzes of Lrp6 mutants showed a subtle increase in Th+ neurons, suggesting that similar to the liver, Mest may influence Wnt signaling through Lrp6. The exact mechanisms of the large defect in Mest ablated animals remains to be elucidated, although the restricted expression in the SNc subset may indicate the specific loss of these neurons. The fact that the overall involvement of modulated Wnt signaling may affect the subset specification of mdDA neurons was recently underscored by the fact that Dkk3 mutants also show a specific loss of rostrolateral mdDA neurons (Fukusumi et al., 2015). In conclusion, Wnt signaling together with it's modulators may form a essential post-mitotic signal that segregates mdDA neurons in the separate subsets as also suggested above.

### 4.6. NeuroD1 and NeuroD6 are essential for the generation of a subset of VTA neurons.

Based on initial gene expression comparison studies performed on human PD patients and controls, NeuroD6 was identified to be enriched in VTA neurons (Chung et al., 2005). This pointed toward a possible role of NeuroD proteins and NeuroD6 specifically in the generation of VTA subsets (Khan et al., 2017). Initial expression analysis showed NueroD6 is present in a VTA subgroup identified by Otx2, Calbindin1 and Ahd2 (Aldh1a1) expression as earlier defined (Di Salvio et al., 2010a). Further refinement of the subset was performed by co-staining a NeuroD6-Cre driven Yfp-reporter line with Grp, a peptide earlier to be found enriched in VTA neurons (Chung et al., 2005) and later mapped to the most ventral rim of the caudal VTA (Smits et al., 2013). These data indicated that NeuroD6 overlaps completely with the expression of Grp, thereby possibly defining this specific VTA subset. Subsequent full ablation of NeuroD6 led to the loss of centrally located mdDA neurons to about 30% at P3. Whether this includes all Grp expression neurons has not been established. In the same mutant the projection area was analyzed and it was apparent that Th-positive connections to the lateral septum were lost probably as a consequence of initial neuronal loss. This suggestions was substantiated by using retrograde tracing from the lateral septum which labeled the Ypf-positive neurons in the NeuroD6-Cre driven Yfp-reporter line. The data suggested that other NeuroD factors expressed in the region may also be involved in the generation of mdDA neurons (Khan et al., 2017). Expression analysis suggested that NeuronD1 was the candidate of choice next to NeuroD6 and ablation studies indicated that NeuroD1 is at least as important as NeuroD6 in generating the VTA neuronal subset as defined above. Taken together, this elegant study (Khan et al., 2017) clearly shows the importance of NeuroD proteins in the generation of a Grp positive VTA neuronal subset with consequences for the innervation of the lateral septum.

## 5. Conclusions

The first data that hinted toward subset specific molecular coding involved in the generation of mdDA neuronal subsets was published at least a decade ago (Smidt et al., 2004). Subsequent papers have theorized on the possible pool size of subsets based on the presumed origin of mdDA neurons within the mesodiencephalon (Smits et al., 2006). The current situation suggests that those original ideas were not completely accurate although the concept and existence of multiple mdDA subsets has now firmly been established. From initial gene expression studies and more recent single cell transcriptome data the presence of at least 5 subgroups has been established in mice. Not all of these groups contain unique genes, but merely rely on the unique combination of genes to distinguish them from each other. In humans the situation is similar although some genes defining the specific subsets do differ. During development the overall idea is that 2–3 subgroups initially exist and that these further develop into the mature 5–6 groups. The molecular coding underlying the initial distinction during development can be explained through the intersectional signaling of factors like Pitx3, En1, Otx2, Lmx1a, NeuroD1/6, and Sox6. The further distinctions toward the adult neuronal pool is at this moment not exactly clear and might depend on additional signaling from the region were these neurons mature. Data that describe the influence of Wnt signaling modulators as Rspo2, Mest and Lrp6 do suggest that Wnt signaling in postmitotic mdDA neurons may have a crucial role in crealting the adult full diversity. Taken together, It is clearly established that in the mammalian dopamine mdDA system a large diversity exists that is reflected in physiological parameters of the respective dopaminergic subsets. Finally, the knowledge of the molecular make-up of mdDA neuronal subsets, as they do exist even within the SNc, is essential for understanding their specific vulnerability and may form the basis for directed cell replacement therapies, evaluated as an ultimate treatment for Parkinson's disease.

## Author contributions

The author confirms being the sole contributor of this work and approved it for publication.

### Conflict of interest statement

The author declares that the research was conducted in the absence of any commercial or financial relationships that could be construed as a potential conflict of interest.
